# Validity of a portable spirometer in the communities of China

**DOI:** 10.1186/s12890-022-01872-9

**Published:** 2022-03-05

**Authors:** Shan Xiao, Fan Wu, Zihui Wang, Jianmin Chen, Huajing Yang, Youlan Zheng, Zhishan Deng, Jieqi Peng, Xiang Wen, Peiyu Huang, Cuiqiong Dai, Lifei Lu, Ningning Zhao, Pixin Ran, Yumin Zhou

**Affiliations:** 1 National Center for Respiratory Medicine, State Key Laboratory of Respiratory Disease, National Clinical Research Center for Respiratory Disease, Guangzhou Institute of Respiratory Health, The First Affiliated Hospital of Guangzhou Medical University, 151 Yanjiang Xi Road, Guangzhou, China; 2 Guangzhou Changhu Medical Devices Co, Ltd., Guangzhou, China

**Keywords:** Portable spirometry, Chronic airway diseases, Consistency, Validity

## Abstract

**Background:**

The lack of simple and affordable spirometry has led to the missed and delayed diagnoses of chronic respiratory diseases in communities. The PUS201P is a portable spirometry developed to solve this problem.

**Objective:**

We aimed to verify the consistency of the PUS201P spirometer with conventional Jaeger spirometer.

**Methods:**

In this cross-sectional study, we randomly recruited 202 subjects aged > 40 years. Testing with the portable spirometry and conventional spirometry were performed on all participants. We compared forced expiratory volume in one second (FEV_1_), forced vital capacity (FVC), FEV_1_/FVC measured by the PUS201P device with the conventional spirometer. Pearson correlation coefficient and Interclass Correlation Coefficient (ICC) were assessed to confirm the consistency of the measures from two instruments. Bland–Altman graph was created to assess the agreement of the measures from two devices.

**Results:**

202 participants were included in this study. The ICC on FEV_1_, FVC, FEV_1_/FVC measured by the portable spirometer and the conventional spirometer were 0.95 (95% confidence interval [CI]: 0.94–0.96), 0.92 (95% CI: 0.90–0.94], 0.93 (95% CI: 0.91–0.95), respectively. The Bland–Altman plots showed that the mean difference between the measures from two spirometers are always located in the 95% limits of agreement.

**Conclusions:**

Our results support that the measures from the portable spirometer and the conventional spirometer have a good agreement and reproducibility. And the portable spirometer is a reliable tool to screen and diagnose chronic airway diseases in the primary care settings.

**Supplementary Information:**

The online version contains supplementary material available at 10.1186/s12890-022-01872-9.

## Background

Spirometry is the fundament of respiratory function test and is the key to diagnosing and supervising the most common chronic respiratory diseases (CRD) [1], and is recommended in practice guidelines for the diagnosis and management of chronic obstructive pulmonary disease (COPD) and asthma [2, 3]. What’s more, as a diagnostic test, spirometry is a reliable, simple, non-invasive, safe, and non-expensive procedure for the detection of airflow obstruction [4]. Therefore, the spirometry as a currently available tool for the early diagnosis of COPD and asthma is particularly important. Epidemiological data show that CRD has contributed to the magnitude of the non-fatal health burden globally [5]. COPD is a worldwide public health challenge because of its high prevalence and related disability and mortality [6–9]. The Global Burden of Disease Study estimated that 3.2 million people has died from COPD worldwide in 2015 [7]. In China, COPD was the third leading cause of death and accounted for more than 0.9 million deaths in 2013 [10]. Asthma is also one of the most common CRD and the global prevalence of self-reported, doctor-diagnosed asthma in adults is 4.3% (95% confidence interval [CI]: 4.2–4.4) [11]. The United States healthcare system estimated that the total asthma related costs was continued to rise, and jumped from USD 53 billion for 2007 to USD 56 billion for 2009, and most recently USD 82 billion in 2013 [12, 13].

Considering the high prevalence and high mortality of CRD, it is important to promote spirometry. However, the respiratory function test is not widely used in the primary care settings. ERS (European Respiratory Society) guidelines quote evidence that up to 75% of COPD patients in Europe remain under-diagnosed [14]. And a large population-based survey reported that Chinese patients who have COPD, only 6.5% have been tested with spirometry [15]. A previous study noted that only 60% had been diagnosed with asthma, and less than 10% had objective assessment of airway function [16]. As a consequence of the lack of simple and affordable spirometry the missed diagnosis of CRD is common. Many CRD patients are usually diagnosed when their condition is very serious. CRD underdiagnosis delays the treatment opportunity. The Burden of Lung Disease estimates of COPD underdiagnosis are substantially higher than those reported for high blood pressure, hypercholesterolaemia, and other similar disorders [4]. Therefore, early diagnosis of CRD is a daunting task in primary care settings. This can be attributed to several factors, including heavy and expensive spirometry equipment, complex program, maintenance charge and professional training for the reliable quality of test and interpretation, influence the accessibility of conventional spirometry [17, 18]. As a result, many primary care physicians require their patients to medical center for spirometric evaluation [19], and the financial burdens of patients were increased.

In recent years, quite a lot of portable spirometers have emerged on the market, only a part of the products has been accessed in clinical trials [20, 21]. One of the portable spirometry used to detect pulmonary ventilation has a good consistency with the convention spirometer [22]. There are several kinds of spirometry, such as full body plethysmography, fully portable units that are wirelessly connected to mobile phones. One of the more popular methods for evaluating patients with CRD is a clinical-grade, in-office, handheld spirometry solution [23]. At present, there are some evidences to support that handheld spirometer has good sensitivity and specificity to identify airflow limitation compared with standard laboratory-based spirometry [24–29].

**Fig. 1:**
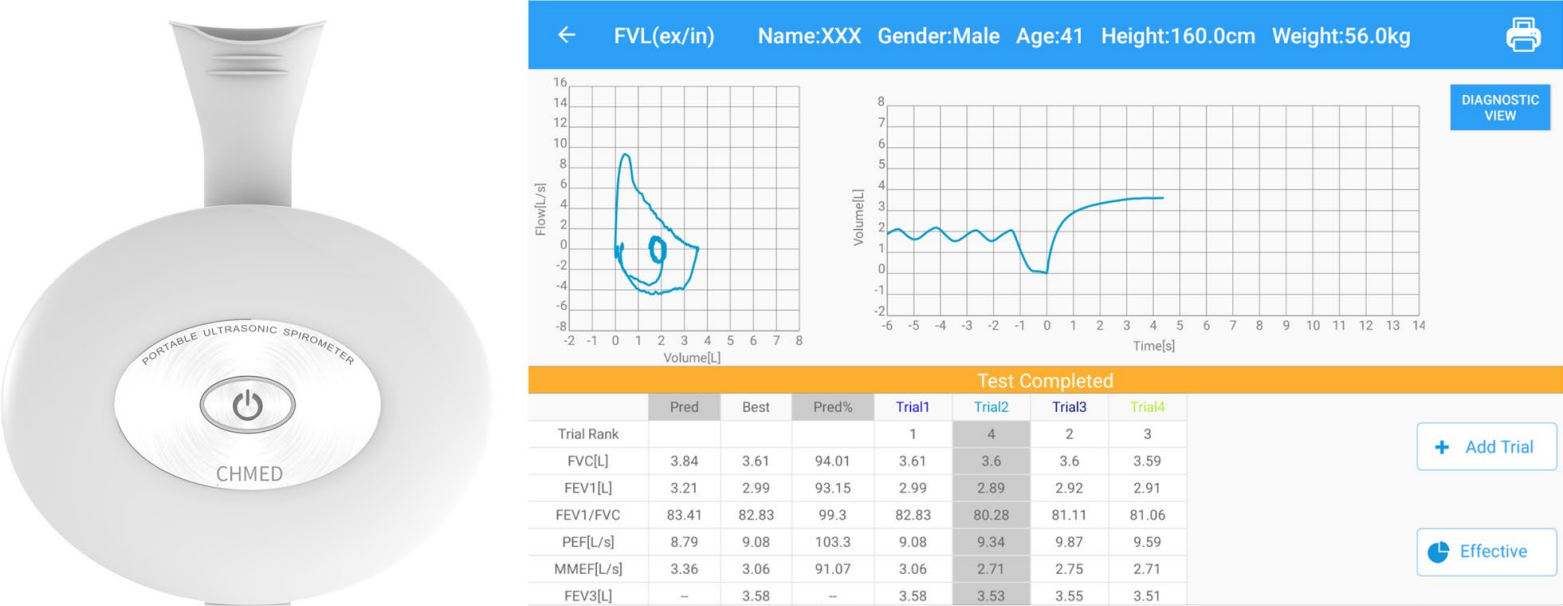
The portable spirometer appearance, work interface and quality control platform. *FVC* forced vital capacity; *FEV*_1_ forced expiratory volume in one second; *PEF* peak expiratory flow; *MMEF* maximum mid-expiratory flow; *FEV*_3_ forced expiratory volume in three seconds

One of the latest devices in China is marketed as PUS201P (Guangzhou Changhu Medical Equipment Co. LTD). The PUS201P is a handheld spirometer does not require regular calibration. It is a portable device connected to a smartphone or tablet computer via Bluetooth and verified by the medical device registration certificate of China. The smart spirometric indices included forced vital capacity (FVC), forced expiratory volume in 1 s (FEV_1_), forced expiratory volume in three seconds (FEV_3_), forced expiratory volume in six seconds (FEV_6_), FEV_1_/FVC, FEV_3_/FVC, FEV_6_/FVC, Peak expiratory flow (PEF), Maximum mid-expiratory flow (MMEF), forced expiratory flow after 25% of FVC has been exhaled (FEF25), forced expiratory flow after 50% of FVC has been exhaled (FEF50), forced expiratory flow after 75% of FVC has been exhaled (FEF75). The specific product appearance, work interface and quality control platform are shown in the Fig. 1.

The purpose of our research is to verify the consistency of the portable spirometer with traditional Jaeger spirometer, and whether the portable spirometer can be used in the screening and diagnosis of chronic airway diseases in primary care settings.

## Methods

We conducted a cross-sectional study in Guangdong Province, China. We randomly recruited 202 subjects aged > 40 years from June 2020 to October 2020. Patients who had any of the contraindications to make spirometry listed in the American Thoracic Society (ATS) and ERS guidelines were excluded: chest or abdominal pain of any cause, oral or facial pain exacerbated by a mouthpiece, stress incontinence, dementia or unconsciousness state [30]. We also excluded patients who did not provide informed consent and those who required more than 8 maneuvers in order to be able to meet reproducibility criteria.

Every participant was performed pulmonary function test with the conventional spirometer and the portable spirometer. The Jaeger spirometer needs linear verification, therefore, we did daily calibration with a 3 L syringe. The PUS201P does not need linear verification. The order of testing for the same patient was randomized and conducted in single-blind fashion, meaning the subjects were unaware as to which machine was under study [23]. Each patient was operated by the same physician-technician. Operations with both equipment were carried out by trained personnel in a standardized way, according to the ATS/ERS guidelines [31]. The acceptability criteria are a satisfactory start of test and a satisfactory end: 1) Start without hesitation, or back extrapolated volume < 5% of FVC or 0.150 L, 2) without coughing during the first second of the operation, 3) without early termination of expiration, 4) curve shows no change in volume for 1 s or exhalation times of > 6 s [31]. We did all spirometric manoeuvres with the participant in a seated position, wearing a nose clip, and using a disposable mouthpiece [32]. Every day we would store test results in the spirometer and downloaded them daily to a central computer system. An expert panel did quality control, excluded tests with poor quality [31]. For each spirometry were recorded the below parameters: FVC, FEV_1_, FEV_3_, FEV_6_, FEV_1_/FVC, FEV_3_/FVC, FEV_6_/FVC, PEF, FEF25, FEF50 and FEF75. Subjects with a pre-bronchodilator FEV_1_/FVC < 70% were defined as spirometry-defined COPD, and healthy control was defined as pre-bronchodilator FEV_1_/FVC ≥ 70%.

All subjects who had a successful spirometry test were required to complete a comprehensive questionnaire to collect data on age, sex, and smoking status. The questionnaire used in this study was a revised form of the international BOLD study [33]. The questionnaire covered demographic data, respiratory symptoms/disease, comorbidities, health care use, activity limitation, potential risk factors for COPD, and the modified Medical Research Council dyspnea scale (mMRC, scores range from 0 to 4, with higher scores indicating more severe breathlessness), the changes in the COPD Assessment Test (CAT) score (scores range from 0 to 40, with higher scores indicating more severe disease score and health status). We defined current smoking as having smoked 100 cigarettes in one’s lifetime and currently smoking cigarettes. We defined passive smoking as inhalation of smoke by nonsmokers who lived with smokers.

The participants were made fully aware of the purpose of study, and all subjects have signed the informed consent before the examination. The study was approved of The Ethics Commission of the First Affiliated Hospital of Guangzhou Medical University approved the study (No. 2018-53).

### Statistical analysis

The qualitative variables were expressed by their absolute value and their percentage, and the quantitative variables are expressed as means and standard deviations (SD). The quality of a medical device was assessed by its consistency with the gold standard (i.e., conventional spirometry). The Bland–Altman method is the preferred method to assess agreement between medical instruments measuring continuous variables [34–36]. Therefore, Bland–Altman method was used to describe the bias between the mean differences for the values obtained by the two devices with Medcalc software. The Pearson correlation coefficient and the Interclass Correlation Coefficient (ICC) were assessed to analyze the consistency of the two instruments. ICC and The Pearson correlation coefficient analyses were conducted using IBM SPSS statistics, version 25. The scatter plot of correlation graphs obtained by the two spirometric devices with GraphPad Prism v. 8.0.2 (GraphPad Software, San Diego California, USA).

## Results

A total of 202 subjects have randomly recruited from a cohort of people with chronic respiratory diseases in Guangdong Province, China. The baseline demographic characteristics of participants who completed questionnaires are showed in Table 1. The mean age of the volunteers was 58.2 years, and 98 (48.5%) participants have never smoked, 78 (38.6%) were current smoking, 26 (12.9%) were former smoking. 198 (98.0%) people had a mMRC score < 2, 14 of 202 subjects have got a CAT score of 10 or more.

**Table 1:** Characteristics of the subjects

Characteristics	Patients (N = 202)
Age- year	58.24 ± 9.72
Sex-no. (%)	
Male sex	133 (65.84)
Female sex	69 (34.16)
Body-mass index	23.47 ± 3.26
Smoking status	
Never smoked	98 (48.52)
Current smoking	78 (38.61)
Former smoking	26 (12.87)
mMRC dyspnea scale score	
Distribution-no. (%)	
< 2	198 (98.02)
≥ 2	4 (1.98)
CAT score	
Distribution-no. (%)	
< 10	188 (93.01)
≥ 10	14 (6.99)
Spirometry-defined COPD	55 (27.2)

*mMRC* modified Medical Research Council; *CAT* COPD Assessment Test; *COPD* chronic obstructive pulmonary disease

In this study, spirometry was performed on every participant with the conventional spirometer and the simple spirometer. For the sake of obtain representative results, the order of testing was randomized. The following spirometric parameters were recorded for all participants from two spirometers: FVC, FEV_1_, FEV_1_/FVC, PEF, MMEF, FEF25, FEF50 and FEF75. The key spirometric parameters of 202 patients measured with two spirometers were showed in Table 2.

**Table 2:** Key spirometric parameters of 202 patients, with both spirometers: (1) Jaeger spirometer and (2) PUS201P spirometer

Parameters	Group	Mean	SD	Max–Min
FVC, L	Jaeger	3.21	0.70	4.86–1.50
	PUS201P	3.13	0.67	4.76–1.56
FEV_1_, L	Jaeger	2.36	0.63	3.81–0.73
	PUS201P	2.31	0.61	3.97–0.73
FEV_1_/FVC, %	Jaeger	73.55	10.85	92.79–31.85
	PUS201P	73.44	10.34	92.55–32.52
MMEF, L/s	Jaeger	1.85	0.99	6.34–0.19
	PUS201P	1.96	0.96	5.84–0.22
PEF, L/s	Jaeger	6.00	1.88	11.21–2.09
	PUS201P	5.82	1.88	11.51–1.96
FEF25, L/s	Jaeger	5.10	1.95	10.14–0.58
	PUS201P	5.00	1.91	9.79–0.60
FEF50, L/s	Jaeger	2.56	1.28	7.33–0.25
	PUS201P	2.63	1.25	7.63–0.22
FEF75, L/s	Jaeger	0.61	0.41	2.88–0.07
	PUS201P	0.69	0.40	2.69–0.08

*FEV*_1_ forced expiratory volume in one second; *FVC* forced vital capacity; *MMEF* maximum mid-expiratory flow; *PEF* peak expiratory flow; *FEF25* forced expiratory flow after 25% of FVC has been exhaled; *FEF50* forced expiratory flow after 50% of FVC has been exhaled; *FEF75* forced expiratory flow after 75% of FVC has been exhaled

We adapted Pearson correlation coefficients and ICC to evaluate the concordance and correlation between the two devices. Table 3 showed that the metrics (Pearson correlation and ICC) of all parameters have significant (*p* < 0.001). The metrics of several key parameters (FEV_1_, FVC, FEV_1_/FVC) were greater than 0.92. All parameters had great concordance and correlation between the two spirometers in spirometry-defined COPD or healthy control subjects (Additional file 1: Table S1 and Table S2).

**Table 3:** Pearson correlation coefficients and intraclass correlation coefficients (ICC) between the spirometric values obtained with the two spirometers, for the entire dataset (202 patients)

	Pearson correlation	*p* value	ICC (95%CI)	*p* value
FEV_1_, L	0.951	< 0.001	0.951 (0.935–0.962)	< 0.001
FVC, L	0.925	< 0.001	0.924 (0.901–0.942)	< 0.001
FEV_1_/FVC, %	0.934	< 0.001	0.933 (0.913–0.949)	< 0.001
MMEF, L/s	0.864	< 0.001	0.863 (0.824–0.895)	< 0.001
PEF, L/s	0.872	< 0.001	0.875 (0.838–0.904)	< 0.001
FEF_25_, L/s	0.913	< 0.001	0.913 (0.887–0.933)	< 0.001
FEF_50_, L/s	0.888	< 0.001	0.888 (0.855–0.914)	< 0.001
FEF_75_, L/s	0.774	< 0.001	0.774 (0.712–0.824)	< 0.001

*CI* Confidence interval; *FEV*_1_ forced expiratory volume in one second; *FVC* forced vital capacity; *MMEF* maximum mid-expiratory flow; *PEF* peak expiratory flow; *FEF25* forced expiratory flow after 25% of FVC has been exhaled; *FEF50* forced expiratory flow after 50% of FVC has been exhaled; *FEF75* forced expiratory flow after 75% of FVC has been exhaled

**Fig. 2:**
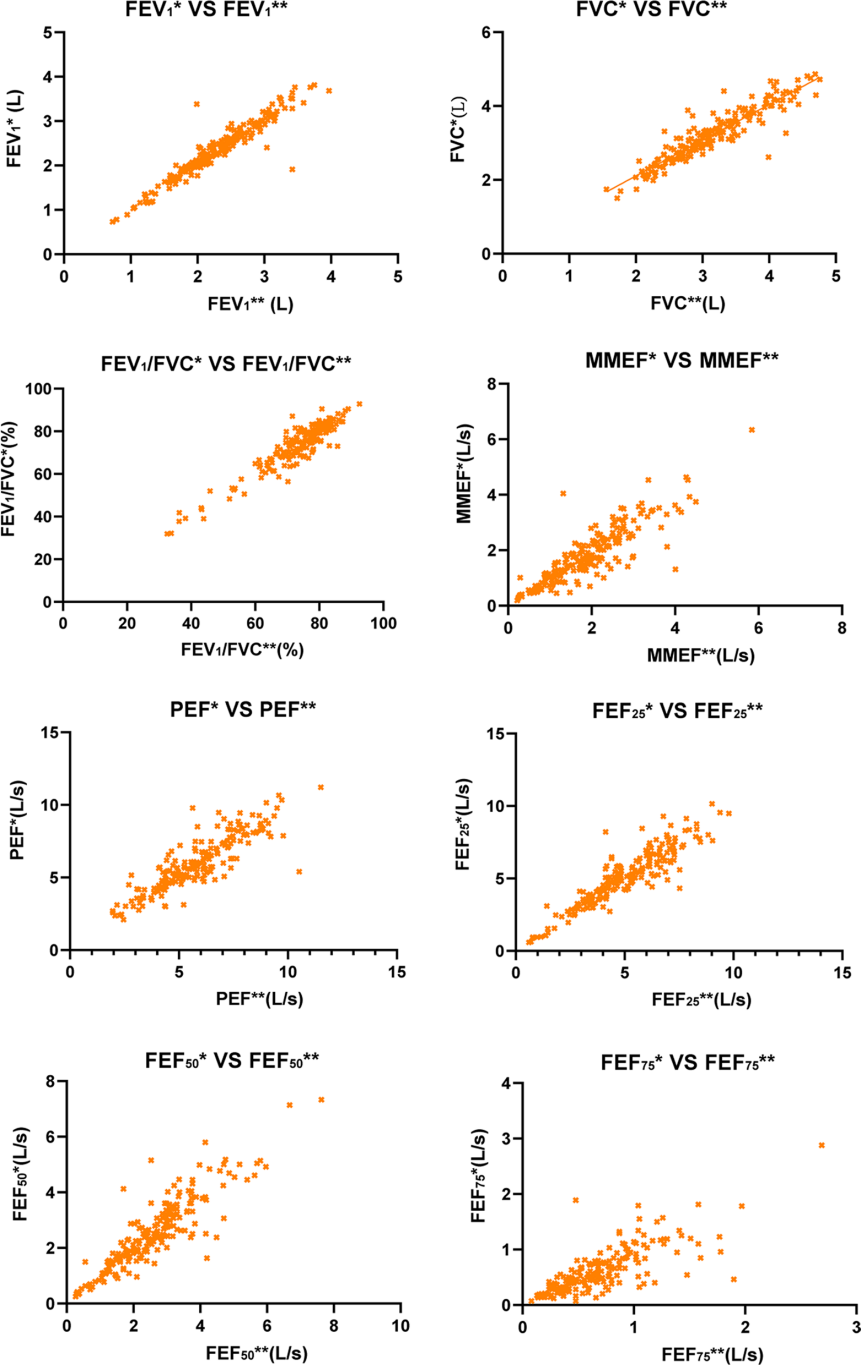
Correlation plots between the values obtained from the two spirometers, for the spirometric parameters considered in this research. *FEV*_1_ forced expiratory volume in one second; *FVC* forced vital capacity; *MMEF* maximum mid-expiratory flow; *PEF* peak expiratory flow; *FEF25* forced expiratory flow after 25% of FVC has been exhaled; *FEF50* forced expiratory flow after 50% of FVC has been exhaled; *FEF75* forced expiratory flow after 75% of FVC has been exhaled. *Note* * = measured by the portable spirometer; ** = measured by the conventional spirometer

A strong linear relationship was found between two devices in all parameters (Fig. 2). As exhibited in the plots, there was significant agreement between the two spirometers. The correlation of FEV_1_ between the portable spirometer and Jaeger spirometer was the strongest (r = 0.904, *p* < 0.001), while the correlation of MMFE, PEF, FEF75 was slightly weak.

**Fig. 3:**
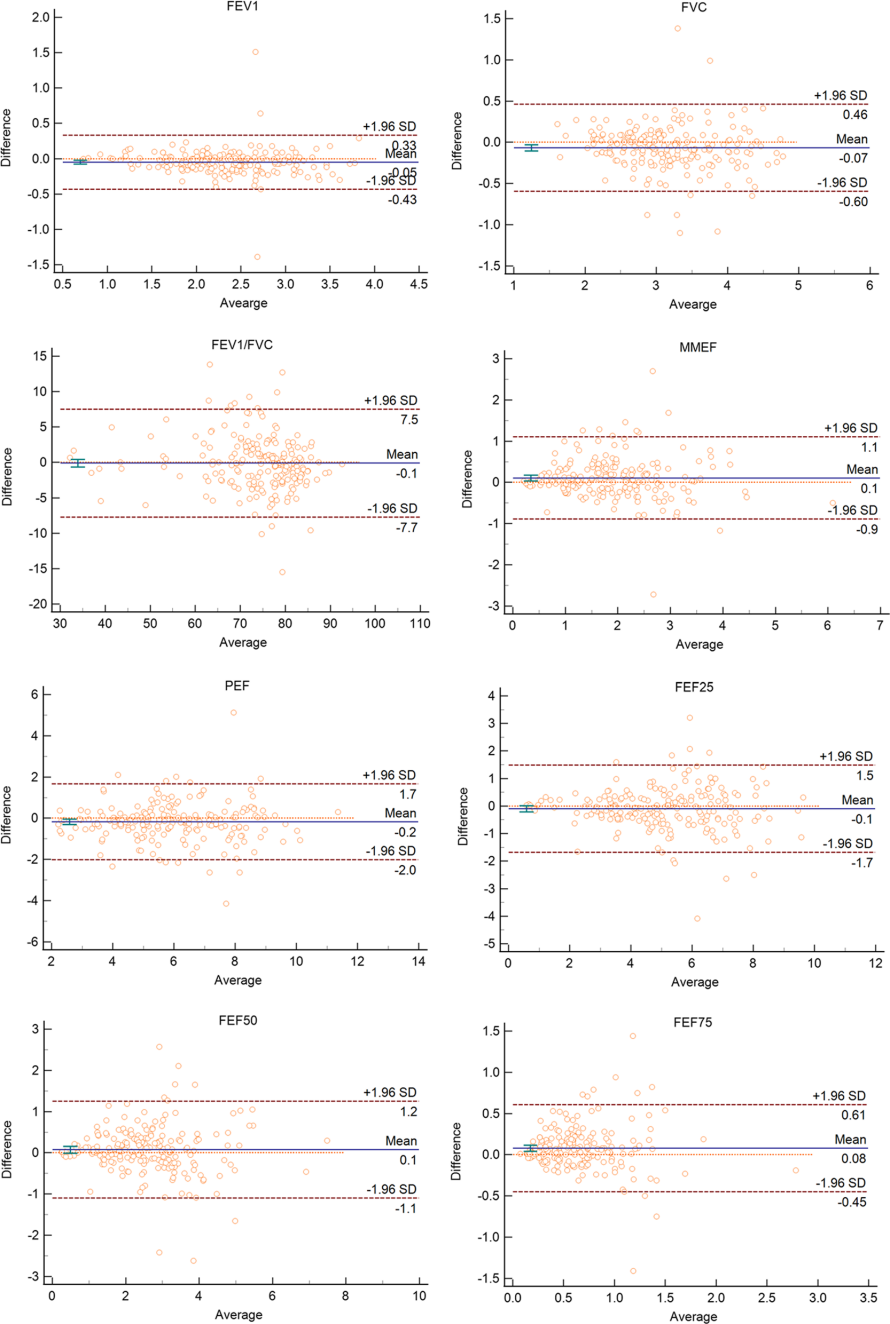
Bland–Altman plots for the evaluated spirometric parameters: FEV_1_, FVC, FEV_1_/FVC, MMEF, PEF, FEF25, FEF50, FEF75. Dashed lines represent the mean difference between measurements and dotted lines the 95% limits of agreement. *FEV*_1_ forced expiratory volume in one second; *FVC* forced vital capacity; *MMEF* maximum mid-expiratory flow; *PEF* peak expiratory flow; *FEF25* forced expiratory flow after 25% of FVC has been exhaled; *FEF50* forced expiratory flow after 50% of FVC has been exhaled; *FEF75* forced expiratory flow after 75% of FVC has been exhaled. *Note* * = measured by the portable spirometer; ** = measured by the conventional spirometer

The Bland–Altman plot was drawn to display the mean difference or bias and 95% limits of agreement (95%LoA, ± 1.96 SD) between devices for each value measured (Fig. 3). In these plots, we can find that most of the mean difference were located in the 95%LoA. It can be supported that the results of the two devices have a good consistent.

## Discussion

Aimed to assess whether results from spirometry with the portable spirometer is reliable. The results of our study indicated that the validity and quality of spirometric indices by the portable spirometer is satisfactory in comparison with the conventional spirometer (Jaeger spirometer). Spirometric measurements with the portable spirometer presented great agreement (calculated by Pearson correlation coefficients and ICC) and reproducibility (in the Bland–Altman plots) with the Jaeger spirometer. Great ICC values were obtained for all measured variables consistently.

The ICC and Pearson correlation coefficients values between two devices parameters reflected great reliability. The metrics of certain key parameters (FEV_1_, FVC, FEV_1_/FVC) were greater than 0.92. Although ICC values of MMEF, PEF, FEF75 were slightly lower but remained significant (> 0.75), which was in line with previous findings [25, 37–39]. The participants did not exert continuous force to reach the flow limit during the whole process of FVC measurement may affect this result.

Spirometry is recommended as a diagnostic and therapeutic tool in primary care setting, and it should be promoted as a method of facilitating accurate diagnosis of chronic obstructive pulmonary disease [40]. Unfortunately, spirometry is invaluable as a screening test of general respiratory health in the same way that blood pressure provides important information about general cardiovascular health [31]. There's an evidence showed spirometry remains largely underused in primary care [17]. This may be attributable to several factors, including large disparities in health care resources, negligence of primary care physicians and the patient [23]. So, it is important to apply a new medical device that is cheap, easy to operate, and as accurate as the gold standard method.

At present, there are several portable spirometers validated to date, such as the Air Next spirometer and the EasyOne spirometer [41, 42]. For example, the Air Next spirometer is the disposable turbines a pre-calibrated tachograph and does not need calibration [41]. Our portable spirometer is a handheld spirometer equipped with an ultrasonic sensor to measure air flow, and the accuracy of this portable spirometer is not influenced by daily temperature and humidity. Our portable spirometer has a great application prospect. It has high consistency with conventional spirometer, lower instrument cost, smaller size for easy handling, less effort to perform the test, improved ease of calibration checks.

This is the first study to assess the validity and safety of the portable spirometer device with conventional spirometer in Chinese community. All participants were recruited from community screening. And there were many healthy subjects, not only the patients included in our study, which were different from previous study [22, 23, 41]. Our research focused on the potential application of the portable pulmonary function instrument in the communities. From a methodological point of view, randomization of the order for the conventional spirometer and the portable spirometer is important to come out with a reliable result [43]. Another crucial concern on the utilization of portable spirometers is the cost of instruments. Our smart spirometer is much cheaper than traditional spirometer.

There are several limitations in our study. First, the study was designed to determine the agreement and validity of a portable spirometer, thus the bronchodilator reversibility testing was not routinely performed as part of our procedure. Second, the measurement of Jaeger pulmonary function instrument is regarded as the "standard" artificially, however, there are also systematic errors in the conventional spirometry. In addition to, our portable spirometry can only detect pulmonary ventilation function, but not diffusion function and impulse oscillometry. Although the limitations of portable spirometry were significant, spirometry in confirming and excluding obstructive airway disease is essential for early diagnosis and treatment of CRD in the primary care [44].

## Conclusion

Our findings suggested that spirometric measurements with the PUS201P spirometer has a good agreement and reproducibility with the Jaeger spirometer. And the PUS201P is a reliable tool to screen and diagnosis chronic respiratory diseases in primary care setting.

## Supplementary Information

**Additional file 1**.** Table S1**. COPD group’s Pearson correlation coefficients between the spirometric values obtained with the two spirometers, for the entire dataset (55 patients).** Table S2**. Health group’s Pearson correlation coefficients between the spirometric values obtained with the two spirometers, for the entire dataset (147 patients).

## Data Availability

The datasets used and/or analyzed during the current study are available from the corresponding author on reasonable request.

## Abbreviations

CI: Confidence interval
CRD: Chronic respiratory disease
COPD: Chronic obstructive pulmonary disease
FEV_1_: Forced expiratory volume in one second
FEV_3_: Forced expiratory volume in three seconds
FEV_6_: Forced expiratory volume in six seconds
FVC: Forced vital capacity
MMEF: Maximum mid-expiratory flow
L/s: Litres per second
PEF: Peak expiratory flow
FEF25: Forced expiratory flow after 25% of FVC has been exhaled
FEF50: Forced expiratory flow after 50% of FVC has been exhaled
FEF75: Forced expiratory flow after 75% of FVC has been exhaled
CAT: COPD assessment test
mMRC: modified Medical Research Council
ATS: American Thoracic Society
ERS: European Respiratory Society
