# Supplementary material for: Validity of a portable spirometer in the communities of China
Source: BMC Pulm Med. 2022 Mar 5;22:80. doi: 10.1186/s12890-022-01872-9 (PMC8898436; doi:10.1186/s12890-022-01872-9)
Supplement: Supplementary file 1 — Additional file 1. Table S1. COPD group’s Pearson correlation coefficients between the spirometric values obtained with the two spirometers, for the entire dataset (55 patients). Table S2. Health group’s Pearson correlation coefficients between the spirometric values obtained with the two spirometers, for the entire dataset (147 patients). [file 12890_2022_1872_MOESM1_ESM.doc]

**Table S1.** COPD group’s Pearson correlation coefficients between the spirometric values obtained with the two spirometers, for the entire dataset (55 patients).

| | **Pearson correlation** | **P value** | **ICC (95%CI)** | **P value** |  | | --- | --- | --- | --- | --- | | **FEV_1_, L** | 0.985 | ＜0.001 | 0.982(0.970-0.990) | ＜0.001 | | **FVC, L** | 0.920 | ＜0.001 | 0.910(0.850-0.946) | ＜0.001 | | **FEV_1_/FVC, %** | 0.934 | ＜0.001 | 0.930(0.882-0.958) | ＜0.001 | | **MMEF, L/s** | 0.789 | ＜0.001 | 0.761(0.623-0.853) | ＜0.001 | | **PEF, L/s** | 0.917 | ＜0.001 | 0.917(0.862-0.951) | ＜0.001 | | **FEF_25_, L/s** | 0.981 | ＜0.001 | 0.981(0.967-0.989) | ＜0.001 | | **FEF_50_, L/s** | 0.887 | ＜0.001 | 0.870(0.787-0.922) | ＜0.001 | | **FEF_75_, L/s** | 0.620 | ＜0.001 | 0.581(0.375-0.732) | ＜0.001 | |
| --- | --- | --- | --- | --- | --- | --- | --- | --- | --- | --- | --- | --- | --- | --- | --- | --- | --- | --- | --- | --- | --- | --- | --- | --- | --- | --- | --- | --- | --- | --- | --- | --- | --- | --- | --- | --- | --- | --- | --- | --- | --- | --- | --- | --- | --- |
| Abbreviations: COPD=chronic obstructive pulmonary disease; FEV_1_=forced expiratory volume in one second; FVC=forced vital capacity; MMEF=maximum mid-expiratory flow; PEF=peak expiratory flow; FEF_25_=forced expiratory flow after 25% of FVC has been exhaled; FEF_50_=forced expiratory flow after 50% of FVC has been exhaled; FEF_75_=forced expiratory flow after 75% of FVC has been exhaled. |

**Table S2.** Health group’s Pearson correlation coefficients between the spirometric values obtained with the two spirometers, for the entire dataset (147 patients).

| | **Pearson correlation** | **P value** | **ICC (95%CI)** | **P value** |  | | --- | --- | --- | --- | --- | | **FEV_1_, L** | 0.924 | ＜0.001 | 0.924(0.896-0.945) | ＜0.001 | | **FVC, L** | 0.932 | ＜0.001 | 0.932(0.907-0.950) | ＜0.001 | | **FEV_1_/FVC, %** | 0.736 | ＜0.001 | 0.735(0.651-0.802) | ＜0.001 | | **MMEF, L/s** | 0.802 | ＜0.001 | 0.801(0.735-0.852) | ＜0.001 | | **PEF, L/s** | 0.837 | ＜0.001 | 0.837(0.781-0.879) | ＜0.001 | | **FEF_25_, L/s** | 0.836 | ＜0.001 | 0.836(0.780-0.879) | ＜0.001 | | **FEF_50_, L/s** | 0.825 | ＜0.001 | 0.824(0.765-0.870) | ＜0.001 | | **FEF_75_, L/s** | 0.714 | ＜0.001 | 0.714(0.624-0.785) | ＜0.001 | |
| --- | --- | --- | --- | --- | --- | --- | --- | --- | --- | --- | --- | --- | --- | --- | --- | --- | --- | --- | --- | --- | --- | --- | --- | --- | --- | --- | --- | --- | --- | --- | --- | --- | --- | --- | --- | --- | --- | --- | --- | --- | --- | --- | --- | --- | --- |
| Abbreviations: FEV_1_=forced expiratory volume in one second; FVC=forced vital capacity; MMEF=maximum mid-expiratory flow; PEF=peak expiratory flow; FEF_25_=forced expiratory flow after 25% of FVC has been exhaled; FEF_50_=forced expiratory flow after 50% of FVC has been exhaled; FEF_75_=forced expiratory flow after 75% of FVC has been exhaled. |
